# Dental Nostrums

**Published:** 1897-08

**Authors:** 


### Editorial.

### DENTAL NOSTRUMS.

THE insertion of secret preparations in the advertising pages of dental journals has lately met with much well-merited censure, as reflecting upon the ethical character of those who have permitted the reprehensible breach of those fundamental ethical principles which should be as binding upon the promoters of dental literature as they are upon the individual members of the profession. It may, indeed, be held as a more serious ethical breach by the management of a journal to admit the blatant advertisements of the nostrum vendors than it is on the part of an individual who may weakly permit himself to fall into the careless or indolent use of such preparations.

Those which have been held to be most seriously objectionable are the class designated as local anæsthetics under various proprietary names. These have received the most urgent criticism because of their dangerous nature, as the essential ingredient of each and all of them is a drug which, unless used with great caution and with full knowledge of the percentage of the toxic ingredient, may be followed by serious consequences to health and to life.

What renders these local anæsthetic nostrums most censurable is that they are pretentiously advertised as harmless, when, from their composition, they are far otherwise than safe to use.

Formulas of local anæsthetics, containing cocaine for various purposes in dental practice, with the required care concerning their employment and the necessary treatment to be taken in case of the appearance of toxic effects, have been repeatedly published. This consideration deprives every honorable person of any excuse for using any one of the advertised nostrums of this kind.

This brings forward another aspect of the subject which clearly places the vendors of secret preparations within the characterization of carrying on a fraudulent imposition upon their fellow-men. When it is considered that the proprietors of the above-stated nostrums are using the common knowledge of the profession, which they have derived from its literature, and palm it off at an amazing profit on the public, the ordinary language which should be applied to them becomes too weak to define the dishonesty of their conduct.

This general subject has been under serious and extended discussion before the American Medical Association, wherein the trustees of the journal of the Association defended their action in admitting doubtful advertisements as a source of profit; but as the result of the questionable light in which it placed the trustees, and as compromising the standing of the journal, they reversed their policy, and determined *to accept no advertisement of medical preparations the proprietors of which do not give a formula containing the official or chemic name and quantity of each composing ingredient, to be inserted as a part of the advertisement.*

It therefore becomes the bounden duty of each dental journal laying claim to honorable character to refuse to accept any advertisement which does not fulfil the requirement of the above rule of the journal of the American Medical Association, and the profession would rightly visit its censure upon the dental journals which disregard this righteous policy. L. J.

